# Multifunctional Probiotic and Functional Properties of *Lactiplantibacillus plantarum* LRCC5314, Isolated from Kimchi

**DOI:** 10.4014/jmb.2109.09025

**Published:** 2021-11-06

**Authors:** Seokmin Yoon, Hyeokjun Cho, Yohan Nam, Miri Park, Ahyoung Lim, Jong-Hwa Kim, Jaewoong Park, Wonyong Kim

**Affiliations:** 1 Department of Microbiology, Chung-Ang University College of Medicine, Seoul 06974, Republic of Korea; 2 Lotte R&D Center, Seoul 07594, Republic of Korea

**Keywords:** *Lactiplantibacillus plantarum* LRCC5314, probiotics, multifunctional properties, kimchi

## Abstract

In this study, the survival capacity (acid and bile salt tolerance, and adhesion to gut epithelial cells) and probiotic properties (enzyme activity-inhibition and anti-inflammatory activities, inhibition of adipogenesis, and stress hormone level reduction) of *Lactiplantibacillus plantarum* LRCC5314, isolated from kimchi (Korean traditional fermented cabbage), were investigated. LRCC5314 exhibited very stable survival at ph 2.0 and in 0.2% bile acid with 89.9% adhesion to Caco-2 intestinal epithelial cells after treatment for 2 h. LRCC5314 also inhibited the activities of α-amylase and α-glucosidase, which are involved in elevating postprandial blood glucose levels, by approximately 72.9% and 51.2%, respectively. Treatment of lipopolysaccharide (LPS)-stimulated RAW 264.7 cells with the LRCC5314 lysate decreased the levels of the inflammatory factors nitric oxide, tumor necrosis factor (TNF-α), interleukin (IL)-1β, and interferon-γ by 88.5%, 49.3%, 97.2%, and 99.8%, respectively, relative to those of the cells treated with LPS alone. LRCC5314 also inhibited adipogenesis in differentiating preadipocytes (3T3-L1 cells), showing a 14.7% decrease in lipid droplet levels and a 74.0% decrease in triglyceride levels, as well as distinct reductions in the mRNA expression levels of adiponectin, FAS, PPAR/γ, C/EBPα, TNF-α, and IL-6. Moreover, LRCC5314 reduced the level of cortisol, a hormone with important effect on stress, by approximately 35.6% in H295R cells. *L. plantarum* LRCC5314 is identified as a new probiotic with excellent in vitro multifunctional properties. Subsequent in vivo studies may further demonstrate its potential as a functional food or pharmabiotic.

## Introduction

Probiotics are defined as living microorganisms that provide health benefits to the host when consumed in sufficient amounts, generally by improving the composition of the gut flora [1]. Probiotics act through various mechanisms, including competition with pathogens for nutrients or adhesion sites, degradation of toxins, production of antimicrobial components, and stimulation of both the innate and adaptive immune systems [2-4].

With rapid advances in microbiome analysis technology, substantial research attention has focused on the gut microbiome in various diseases. In particular, the effects of an adequate gut microbiome and probiotic supplementation on type 2 diabetes, obesity, cardiovascular diseases, and a variety of human diseases have recently been recognized [5-7]. Moreover, the number of studies focused on developing more effective probiotics continues to increase. An effective probiotic must be viable, safe, tolerant against the action of bile and gastric juices while passing through the gastrointestinal tract, and capable of colonizing intestinal epithelial cells through adhesion [8, 9].

*Lactiplantibacillus plantarum* (*L. plantarum*) is one of the major lactic acid bacteria (LAB) with diverse and unique probiotic properties [10], along with excellent ability to thrive in harsh environments, such as high acid and bile tolerance and antagonistic action against gut pathogens [11-13]. However, even strains within the same genus have different probiotic properties, exhibiting strain-specific variability [14, 15]. Since not all probiotics may offer health benefits to the host, it is essential to accurately identify its unique probiotic properties, before using any potential probiotic strain [16].

Therefore, the aim of this study was to characterize the basic probiotic properties, including acid and bile tolerance and adhesion to intestinal epithelial cells, of a new *L. plantarum* strain, LRCC5314, which was isolated from kimchi—a traditional Korean fermented food—using in vitro assays. Moreover, other potential probiotic properties of LRCC5314 were explored, including those related to blood glucose, obesity, and stress.

## Materials and Methods

### Screening and Isolation of LRCC5314

LAB were isolated from both kimchi sold in traditional Korean markets and homemade kimchi. The kimchi ingredients included cabbage, salted fish, green onions, and salt, with no separate source of LAB added. After addition of 0.85% sterile saline solution (10× in volume), the kimchi samples were homogenized using a homogenizer (Stomacher, Pro-Media SH-001, ELMEX, Japan) and diluted in sterile saline solution. The diluent (100 μl) was plated on de Man-Rogosa-Sharpe (MRS) agar medium (Difco, USA) containing 0.02% bromocresol purple solution and incubated for 48 h in a 37°C incubator. After incubation, colonies with yellow halos, indicating acid production, were screened and sub-cultured thrice in the same medium for pure isolation. Following pure isolation, LAB isolates were placed in MRS broth containing 50% glycerol as cryoprotectant, and stored at –80°C. Before use, the isolates were sub-cultured three times using MRS agar and broth.

To determine its tolerance and adhesion activity, *L. plantarum* KCTC 3108^T^, and *L. plantarum* KCTC 3099, obtained from the Korean Collection for Type Cultures (KCTC) and seven *L. plantarum* strains, LRCC5193, LRCC5226, LRCC5230, LRCC5273, LRCC5277, LRCC5309, and LRCC5310, isolated from kimchi, were used as reference strains for comparison.

### Identification of Strain LRCC5314

LAB isolates were identified using 16S rRNA gene sequence analysis, and amplification of 16S rRNA was performed according to a previously reported method [17]. The 16S rRNA amplicons were sequenced using a 3730 automatic DNA sequencer (Applied Biosystems, USA), and the acquired sequences were analyzed using the BLAST program of the National Center for Biotechnology Information (NCBI) (https://blast.ncbi.nlm.nih.gov/Blast.cgi). Biochemical properties were analyzed using the API 50 CHL kit, and culture and analysis were performed according to the manufacturer’s instructions (bioMérieux, France). The colonies cultured on MRS agar were inoculated into the API 50 CHL kit and cultured at 37°C, and the strip results interpreted after 24 and 48 h. The results were compared against the BioMerieux database (https://apiweb.biomerieux.com) for simple identification.

### Acid and Bile Tolerance

To determine the rate at which LAB isolates reach the intestines, stability under acidic and bile acid conditions was measured in vitro. For the acid treatment, the seed culture broth was centrifuged (10,000 ×*g*, 4°C, 10 min), after which the supernatant was removed and MRS broth with pH adjusted to 2.0 (1 M HCl), added. The number of viable cells was then counted over night to identify the stability of the bacteria in an acidic environment. To determine the influence of bile acid, MRS broth containing 0.2% bovine bile acid (oxgall) (Sigma, USA) was added to the cell culture broth. Thereafter, the supernatant was removed and the number of viable cells counted, after 16 h incubation at 37°C.

### Adhesion Assay

For the intestinal adhesion assay of LRCC5314, Caco-2 cells obtained from the Korean Cell Line Bank (Korea) were used as previously described [12]. After activating the culture medium, prepared using minimal essential medium (Gibco BRL, USA), fetal bovine serum (FBS) (Hyclone, USA), and penicillin-streptomycin (15140-122, Gibco), Caco-2 cells (5 × 10^4^ cells/well) were inoculated in a 96-well plate. The cells were incubated and maintained at 37°C and 5% CO_2_. The medium was exchanged every two days, and the cells were incubated until a monolayer was formed on the 96-well plate.

The culture broth containing bacterial isolates was centrifuged (12,000 ×*g*, 10 min) to collect the cells, after which 200 μl of the bacterial isolate was added to a 96-well plate containing Caco-2 cells and allowed to react for 2 h in an incubator. Upon reaction completion, the 96-well plate was washed three times with phosphate-buffered saline (PBS), and 0.25% trypsin-ethylenediaminetetraacetic acid was used to harvest the Caco-2 cells. After transferal to a 1.5-ml EP tube, the number of colonies was counted following stepwise dilution. To determine the adhesion rate of bacterial isolates to Caco-2 cells, viable bacterial isolates were counted before the reaction, following the addition of the bacterial strain, and 2 h after the reaction. The adhesion to Caco-2 cells was quantified by the log value of the number of viable cells after the reaction, divided by the log value of the number of viable cells before the reaction.

### Enzyme Inhibitory Activity (α-Amylase and α-Glucosidase)

To measure the inhibition of α-amylase activity, a modified version of the method reported by Xiao *et al*. [18] was used. Distilled water (DW) was used to dilute 11 U/mg of α-amylase (a3403, Sigma) to a concentration of 0.5 mg/ml, which was subsequently mixed with an LRCC5314 sample (1 × 10^8^ CFU) and allowed to react for 10 min at 25°C. Soluble starch was prepared at 1% using 0.02 M sodium phosphate buffer (pH 6.9), which was added to a mixture incubated together with DNS reagent (3,5-dinitrosalicylic acid, sodium potassium tartrate, and sodium hydroxide) prepared using water, and allowed to react for 5 min at 100°C. After 5 min, the mixture was cooled to 25°C and 10 ml of DW added, after which the optical density at 540 nm (OD_540_) was measured using a microplate reader (Infinite M200 Nano-quant; TECAN, Switzerland). OD values were compared between samples treated with only α-amylase and those treated with a mixed sample.

Inhibition of α-glucosidase activity was assessed using a slightly modified version of the method reported by Shai *et al*. [19]. A 15 ml tube with 0.35 U/ml of α-glucosidase (G5003; Sigma), 0.1 M sodium phosphate buffer (pH 7.0), and the LRCC5314 sample (1 × 10^8^ CFU) was incubated for 20 min at 37°C. After incubation, 50 μl of 3 mM p-nitrophenyl-β-D-glucopyranoside was added as the substrate and allowed to react for 20 min at 37°C. The reaction was terminated by adding 1 ml of Na_2_CO_3_ (1 M), and the OD_405_ of the released p-nitrophenol was measured using a microplate reader (Infinite M200 Nano-Quant, TECAN).

### Anti-Inflammatory Activity in RAW 264.7 Cells

The 3-(4,5-dimethylthiazol-2-yl)-2,5-diphenyltetrazolium bromide (MTT) assay was used as a safety assessment to measure the effect of LRCC5314 LAB lysates on the viability of RAW 264.7 cells [20]. RAW 264.7 cells from the American Type Culture Collection (ATCC) (TIB-71; USA) were dispensed into a 24-well plate (5 × 10^5^ cells/well), from which the medium was removed after 24 h of incubation. Subsequently, LRCC5314 lysates (1×10^8^ CFU) were added to fresh Dulbecco’s modified Eagle medium (DMEM) (Invitrogen,) and incubated for 24 h. After incubation, 500 μl of 5 mg/ml MTT (Sigma) was added, and the mixture incubated for 1 h at 37°C. After incubation, the supernatant was removed, 500 μl of dimethyl sulfoxide added, and the OD_590_ measured using a microplate reader (Infinite M200 Nano-quant).

RAW 264.7 cells, which were used to identify the immune modulation effect of LRCC5314, were incubated in DMEM containing 10% FBS and dispersed to a 24-well plate at 5 × 10^5^ cells/well, for incubation at 37°C and 5%CO_2_. To induce inflammation, the positive control group was treated with 0.1 μg/ml of lipopolysaccharide (LPS), and the experimental group, with LPS and LRCC5314 lysate (1 × 10^8^ CFU), followed by incubation for 24 h. Nitric oxide (NO) production in the control and LRCC5314 groups was measured using the Griess reaction [21]. Moreover, the levels of inflammatory cytokines TNF-α, IL-1β, and IFN-γ were analyzed using enzyme-linked immunosorbent assay (ELISA) kits (Bio-Rad, USA) according to the manufacturer’s protocol.

### Anti-Adipocyte Differentiation Activity in 3T3-L1 Cells

The ability of LRCC5314 to inhibit adipocyte differentiation was measured using the method described by Sakuma *et al*. [22] with slight modifications in 3T3-L1 cells (ATCC CL-173). The cells were cultured in DMEM containing 10% bovine calf serum (Sigma-Aldrich) and 1% penicillin/streptomycin (Gibco BRL) at 37°C and 5%CO_2_/95% air supply. To induce the differentiation of 3T3-L1 preadipocytes, the cells (5 × 10^4^ cells/well) were dispensed into a 12-well plate. After 2 d, the medium was replaced with fresh medium, and the cells were allowed to form a complete monolayer on the fourth day. Subsequently, the medium was replaced with 10% FBS-DMEM containing DMI solution to induce differentiation over 2 d. During the following 4 d, the cells were incubated in FBS-DMEM containing only insulin to induce adipocyte differentiation. Finally, adipocyte differentiation was terminated by adding FBS-DMEM alone. In the experimental group, the differentiated 3T3-L1 preadipocytes were treated with LRCC5314 lysate (1 × 10^8^ cells/ml) and incubated every day during the experimental period. Oil Red O (Sigma) was used to stain the lipid droplets produced inside the cells to measure intracellular lipid accumulation, as confirmation of adipocyte differentiation. After removing the medium, the cells were washed twice with PBS and fixed with 10% formalin for 1 h at room temperature. Fixed 3T3-L1 cells were washed twice with PBS and stained with 0.5% Oil Red O solution for 30 min at room temperature. After staining, the dye solution was removed, and the cells were washed twice with DW. After removing the DW, isopropyl alcohol was added to completely dried wells, and the OD_500_ measured using a microplate reader (Infinite M200 Nano-Quant). Moreover, triglyceride (TG) levels and mRNA expression levels of the adipocyte differentiation markers, adiponectin, FAS, PPAR/γ, C/EBPα, TNF-α, and IL-6 were measured according to previously reported methods [22].

### Anti-Stress Activity in H295R Cells

The effect of LRCC5314 on stress hormones was analyzed in H295R cells (ATCC). The cells were cultured in DMEM-F-12 containing 10% FBS, 1% penicillin/streptomycin, and a mixture of insulin, transferrin, and selenium at 37°C and 5% CO_2_/95% air. For identification of anti-stress activity, the cells were seeded in a 24-well plate at 5 × 105 cells/well. Stress was induced by treatment with LPS (10 ng/ml) and simultaneous treatment of LRCC5314 lysate (1 × 10^8^ cells/ml), followed by incubation for 24 h. The supernatant was harvested and stored in a deep freezer at –80°C, until analysis. Cortisol, a stress-related factor, was analyzed using ELISA according to the manufacturer’s instructions (BD, USA).

### Manufacture and Standardization of LRCC5314

LRCC5314 was inoculated into a 1-L fermenter containing sterilized media (4% glucose, 0.3% yeast extract, 0.05% KH_2_PO_4_, 0.02% MgSO_4_, and 0.02% MnSO_4_) and fermented under controlled conditions (37°C, 48 h, pH 6.8± 0.2). The cultured broth was then inoculated into a 100-L fermenter and cultured in the same medium, under conditions as previously described. The cell pellet was collected via centrifugation (15,000 ×*g*, 30 min, 4°C) and suspended in the cryoprotectant solution according to the manufacturer’s protocol. The suspension was then freeze-dried at -80°C for 48 h. The lyophilized LRCC5314 powder was stored at 4°C until further use. These processes were repeated three times to standardize the manufacturing process.

Analysis of the manufactured LRCC5314 powder was performed as follows: moisture content was determined using the Karl Fischer method [12]. Firstly, the sample (0.2 g) was dissolved completely in the working medium, which consisted of dichloromethane and dry methanol in a 1:1 ratio, by stirring for 3 min. Thereafter, the sample was mixed with Karl Fischer reagents (iodine and sulfur dioxide in pyridine/methanol mixture) and the titration allowed to continue until a stable electrometric end point was obtained. The moisture content was then automatically calculated using the titrant volume and sample weight.

Detection of *E. coli* and *Salmonella* sp. was conducted by cultivation of manufactured LRCC5314 powder on enrichment media. Eosin methylene blue (EMB) agar (containing 0.4 g eosin Y, 10.0 g pancreatic digest of gelatin, 10.0 g lactose, 2.0 g dipotassium phosphate, 0.065 g methylene blue, and 15.0 g agar per L) was used to detect *E. coli* [3]. Tetrathionate agar (consisting of 2.5 g proteose peptone, 2.5 g pancreatic digest of casein, 1.0 g oxgall, 30.0 g sodium thiosulfate, and 10.0 g calcium carbonate per L) was used to enrich and detect *Salmonella* sp. [23].

### Statistical Analysis

All data are presented as means ± standard deviation of each experimental sample tested in triplicate. Data were analyzed using analysis of variance and Tukey’s post-hoc test, for multiple pairwise comparisons. The statistical significance of difference was set at *p* < 0.05. All analyses were conducted using GraphPad Software (version 6.0).

## Results

### Acid- Bile Tolerance, Adhesion, and Enzyme Inhibitory Activities of LRCC5314

To sufficiently demonstrate the role of a LAB isolate as a probiotic, it must be able to survive stably in the gastrointestinal tract to achieve intestinal colonization. Accordingly, tolerance of the 263 LAB strains identified in kimchi, to low pH and high bile salt concentrations, was assessed. Among these isolates, LRCC5314 was selected for further analysis. The acid-bile tolerance, adhesion, and enzyme activity-inhibition activities of *L. plantarum*, including LRCC5314, are shown in Table 1. The tolerance rate of LRCC5314 after exposure to a highly acidic buffer (pH 2.0), was approximately 110.4%, but no statistically significant difference was observed. Table 1 shows the results before and after treatment with 0.2% oxgall-a bovine bile acid-demonstrating a survival rate of approximately 101.3% after treatment; however, the difference was not statistically significant.

Adhesion of LAB to intestinal epithelial cells also has a significant influence on their effectiveness as probiotics; higher and longer-lasting adhesion activity is more likely to influence the digestive system, immune system, and gut flora of the host. Caco-2 cells were used to measure the adhesion ability of LRCC5314 to intestinal epithelial cells. As shown in Table 1, the viable cell count of LRCC5314 decreased only slightly after 2 h, with cells demonstrating approximately 89.9% adhesion activity. Moreover, LRCC5314 inhibited α-amylase and α-glucosidase activity by approximately 72.9% and 51.2%, respectively, relative to the control (*p* = 0.0004, *p* = 0.0009, respectively). Resultantly, LRCC5314 showed the highest enzyme activity-inhibition efficacy among the evaluated *L. plantarum* strains.

### Identification of the LRCC5314 Strain

For genomic identification, the 16S rRNA gene of the LRCC5314 strain was amplified through PCR and sent to Macrogen Co. (Korea), for sequencing. The resultant sequence data contained 960 base pairs, which were used in a homology search using the BLASTN program on the NCBI website (blast.ncbi.blm.gov) and reported in the GenBank database (accession number: MW828327). From the results, the strain was identified as *L. plantarum*, with closest resemblance to *L. plantarum* WCFS1 (KC429782). The results of the determination of carbohydrate utilization by LRCC5314, which was performed using the API 50 CHL system, are summarized in Table 2. As shown, LRCC5314 was able to utilize many sugars and sugar-alcohols including glucose, and mannitol, among others. However, LRCC5314 was unable to utilize glycerol, xylose, and starch. It most closely resembled the standard strain, *L. plantarum* 1 (99.9% ID, 0.72 T index), and did not respond to ribose or gentiobiose (92.0% and 98.0% respectively).

### Anti-Inflammatory Activity of LRCC5314

**Fig. 1:**
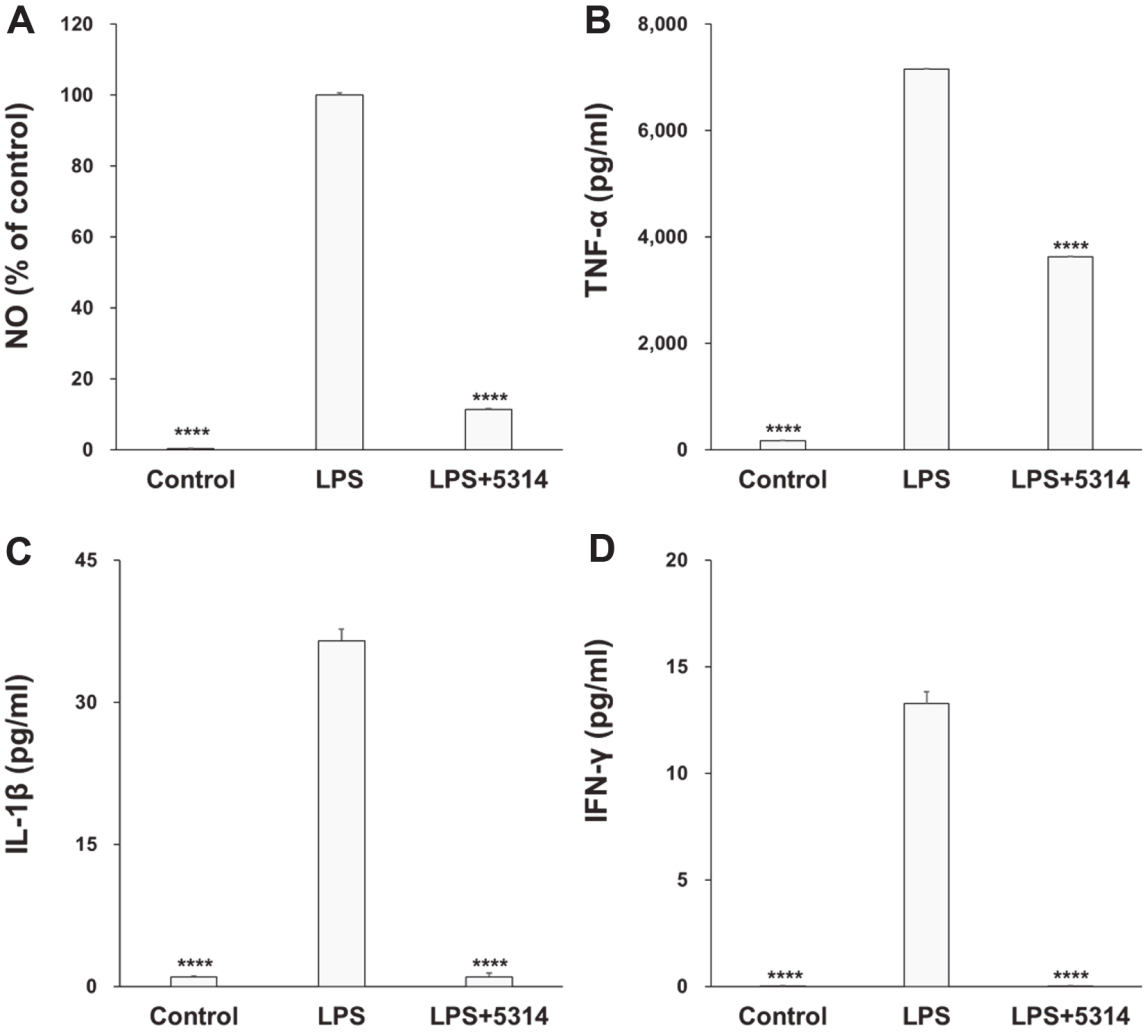
Effects of LRCC5314 on cytokine expression in RAW 264.7 cells. Nitric oxide (NO) production and cytokine levels were assessed by enzyme-linked immunosorbent assay using the cell supernatant after inflammation induction with lipopolysaccharide (LPS). (**A**) NO, (**B**) tumor necrosis factor-α (TNF-α), (**C**) interleukin-1β (IL-1β), and (**D**) interferon-γ (IFN-γ). Results are expressed as the mean ± SE (N = 3). ***p < 0.01.

Results of the MTT assay, performed after treating RAW 264.7 cells with LRCC5314 lysates, showed that cell viability at 1 × 10^8^ colony-forming units (CFU) was not affected, indicating that this isolate is not cytotoxic (*p* = 0.0315). Fig. 1A shows the change in NO levels caused by LRCC5314. Compared to the NO level in RAW 264.7 cells treated with LPS alone, the NO level in cells treated with LRCC5314 lysates decreased to approximately 11.47% (*p* < 0.0001). This indicated that the levels of NO generated by LPS were reduced by approximately 88.5% (*p* < 0.0001). Figs. 1B–1D show the effects of LRCC5314 on the production of the inflammatory cytokines, tumor necrosis factor (TNF)-α, interleukin (IL)-1β, and interferon (IFN)-γ, demonstrating substantial decreases of 49.3%, 97.2%, and 99.8%, respectively (*p* < 0.0001, *p* < 0.0001, *p* < 0.0001 respectively), relative to these levels in cells treated with LPS alone (*p* < 0.0001, *p* < 0.0001, *p* < 0.0001, respectively).

### Anti-Adipocyte Differentiation Activity of LRCC5314

**Fig. 2:**
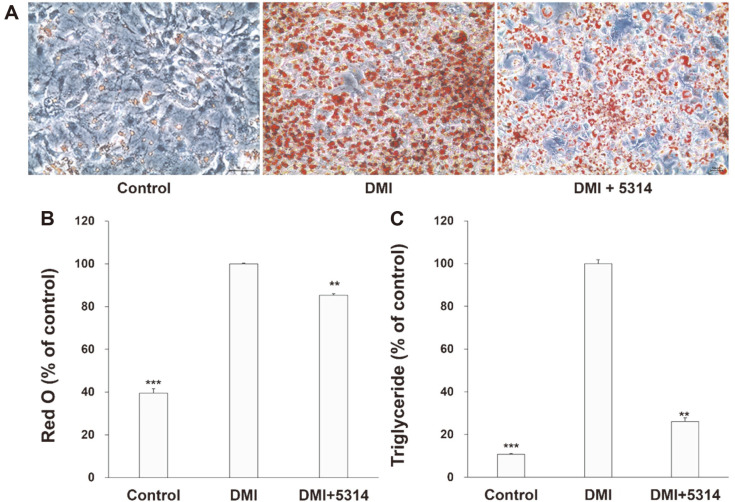
Effects of LRCC5314 on lipid droplet production, adipogenesis, and triglyceride levels in 3T3-L1 adipocytes. (**A**) lipid differentiation was induced by the addition of 1 μM dexamethasone, 0.5 mM 3-isobutyl-1- methylxanthine, and 1 μg/ml insulin (DMI) to 3T3-L1 cells. The lipid droplets stained by Oil Red O (red) were observed under a microscope. (**B**) Oil red-stained lipid droplets were dissolved in isopropanol and the color intensity was measured based on the absorbance value at 450 nm. (**C**) triglyceride levels were assessed by enzyme-linked immunoassay under lipid differentiation induction. Results are expressed as the mean ± SE (N = 3). *** *p* < 0.01.

**Fig. 3:**
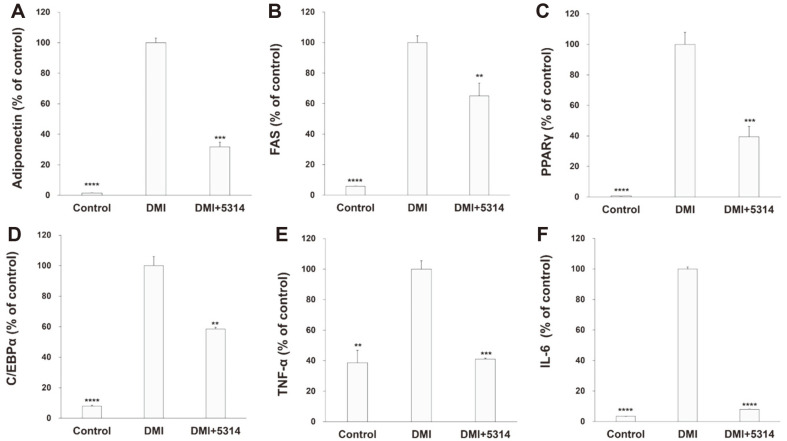
Effects of LRCC5314 on the mRNA expression of adipogenesis-related genes and secretion of inflammatory factors in 3T3-L1 adipocytes. (**A**) Adiponectin, (**B**) FAS, (**C**) PPARγ, (**D**) C/EBPα, (**E**) tumor necrosis factor (TNF)-α, and (**F**) interleukin (IL)-6 measured using real-time polymerase chain reaction and enzyme-linked immunosorbent assay. Results are expressed as the mean ± SE (N = 3). ***p < 0.01.

The changes in adipocyte differentiation activity induced by LRCC5314 lysates were further investigated using 3T3-L1 preadipocytes. Fig. 2A shows the results of Oil Red O staining of lipid droplets in the control and LRCC5314 lysate-treated differentiation-induced 3T3-L1 cells. Many lipid droplets (red dots) appeared within 3T3-L1 cells after differentiation, whereas a clear decrease in lipid droplets was observed after treatment with LRCC5314 lysates. Figs. 2B and 2C show the quantification of Oil Red O staining and measurement of TG concentration, expressed as percentages relative to that of the control group of 3T3-L1 cells treated with only the differentiation medium, which consisted of 1 μM dexamethasone, 0.5 mM 3-isobutyl-1-methylxanthine, and 1 μg/ml insulin (DMI). Treatment with LRCC5314 lysates resulted in an approximate 14.7% decrease in lipid droplets and an approximate 74.0% decrease in the TG concentration (*p* = 0.0004, *p* = 0.0046 respectively). The control group showed approximately 39.4% lipid droplet content and 10.8% TG concentration (*p* = 0.0008, *p* = 0.0003, respectively). Moreover, after treatment with LRCC5314 lysates, the mRNA levels of adipogenesis-related genes, adiponectin, FAS, PPAR/γ, and C/EBPα decreased significantly (*p* = 0.0003, *p* = 0.0007, *p* = 0.0001, *p* = 0.0008, respectively) (Figs. 3A–3D). Increases in TNF-α and IL-6 levels induced by adipogenic differentiation were also prevented by treatment with LRCC5314 lysates, reaching statistically similar levels to those measured before differentiation was induced (*p* = 0.0003, *p* = 0.0001, respectively) (Figs. 3E and 3F).

### Anti-Stress Activity of LRCC5314

**Fig. 4:**
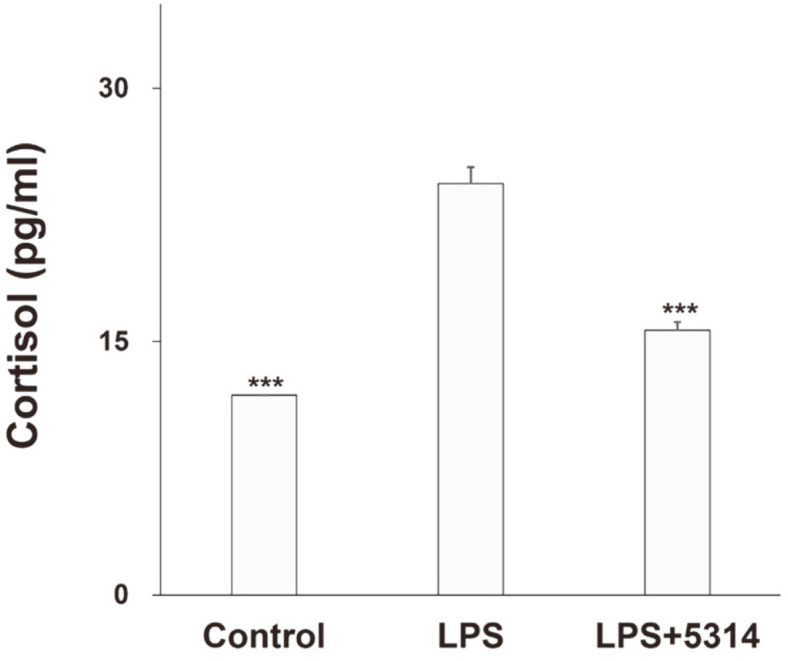
Effects of LRCC5314 on cortisol concentration in H295R cells. Cortisol concentrations were measured by enzyme-linked immunosorbent assay under lipopolysaccharide (LPS) treatment. Results are expressed as the mean ± SE (N = 3). *** *p* < 0.01.

After using LPS to induce stress in H295R cells, the cortisol concentrations produced before and after treatment with LRCC5314 lysates were measured. As shown in Fig. 4, the cortisol level in H295R cells increased significantly after treatment with LPS, but decreased by approximately 35.6% after co-treatment with LRCC5314 lysates (*p* = 0.0008).

### Manufacture and Standardization of LRCC5314

Table 3 shows the results of the standardization of manufactured. More than 3.5 kg of powder was manufactured per lot, and the average amount of manufactured powder was 3.6 kg in three replicate batches. The average moisture content of LRCC5314 was 3.0%, and *E. coli* and *Salmonella* sp. were not detected in any manufactured batch.

## Discussion

Orally-ingested LAB must pass through the stomach, which contains gastric juice and various enzymes, as well as the duodenum-containing bile-before reaching their destination in the intestines, where they exhibit functional efficacy. Saarela *et al*. [24] indicated that excellent survivability in acidic pH and bile conditions, along with adhesion to intestinal epithelial cells are important prerequisites for probiotic action. Although gastric juice typically has a pH of 0.9, the pH in the stomach can increase up to 2 when food is ingested [25]. LAB must also survive in at least 0.3% bile acid conditions to survive in the gastrointestinal tract [26]. Moreover, adhesion to intestinal epithelial cells interacts with the mucosal surface, which facilitates contact with the gut-associated lymphoid tissues that mediate immune effects [27], while also providing a means for the competitive exclusion of pathogenic gut bacteria [28]. Compared with these results, the LRCC5314 in this study showed remarkably improved acid tolerance and adhesion, reaching approximately 90%. The survival rate of LRCC5314 after treatment with an acidic buffer (pH 2.0) and 0.2% oxgall (bovine bile acid) was approximately 110.4% and 101.3%, respectively.

α-Glucosidase, a digestive enzyme, plays a role in the hydrolysis of disaccharides or polysaccharides into monosaccharides for the digestion and absorption of carbohydrates. Therefore, inhibition of α-glucosidase and α-amylase activities suppresses the elevation in postprandial blood glucose levels, thus playing a key role in postprandial blood glucose control in patients with type 2 diabetes and a borderline diabetes status [29]. Kinariwala *et al*. [30] evaluated the inhibitory activity of two L. fermentum strains, M2 and M7, against α-amylase and α-glucosidase; α-amylase and α-glucosidase were inhibited by 63.5–65.3% and by 11.1–13.7%, respectively. In this study, LRCC5314 inhibited the activities of α-amylase and α-glucosidase by approximately 72.9% and 51.2%, respectively, relative to that of the control. Although the results of this study differ from those of previous studies, LRCC5314 demonstrated sufficient potential for the inhibition of enzymatic activity.

Macrophages regulate inflammatory responses by secreting reactive oxygen species such as hydrogen peroxide and NO, and cytokines such as IL-1, IL-6, and TNF-α [31]. LAB can enhance immune function by inducing the phagocytic function of macrophages and activating various immune responses, by stimulating the production of NO and cytokines [32].

Oh *et al*. [33] isolated 22 strains of LAB from feces and found that co-treatment of murine macrophage-RAW 264.7 cells with *Lactobacillus rhamnosus* 4B15 and *Lactobacillus gasseri* 4M13 significantly reduced the LPS-induced increase in the TNF-α, IL-6, IL-1β, and IL-10 levels. Moreover, Weninger and Von Andrian [34] reported that probiotics directly or indirectly reduced the production of TNF-α by inhibiting the production of various inflammatory cytokines such as IL-6 and IL-8. In the present study, RAW 264.7 cells treated with LRCC5314 showed an approximate 88% decrease in NO, along with a 49.3%, 97.2%, and 99.8% decrease in TNF-α, IL-1β, and IFN-γ levels, respectively, under LPS stimulation. These findings suggest that LRCC5314 can enhance immune function by inhibiting the production of inflammatory cytokines and activating the phagocytic function of macrophages.

As the formation of lipid droplets increases, so do TG levels, and lipids gradually accumulate in cells as the enzymes associated with lipid accumulation are activated [35]. Mishra *et al*. [36] reported that application of Oil Red O to differentiated 3T3-L1 preadipocytes produced red-stained lipid droplets, the number of which decreased when *Enterococcus faecalis* AG5 was added. Consistently, in this study, LRCC5314 lysates decreased the number of lipid droplets by approximately 15% and TG levels by approximately 74% in differentiated 3T3-L1 cells, suggesting the potential of this new LAB isolate in ameliorating obesity.

The process of differentiation from preadipocytes to adipocytes involves both morphological changes in lipid droplets, due to lipid accumulation, and molecular and genetic changes such as increased expression of adipocyte-specific protein markers. In addition, the expression of adipogenesis-related transcription factors and the secretion of adipokines show an increasing or decreasing pattern during differentiation [37,38].

Expression of CEBPα and PPARγ is induced in the early stage of differentiation to induce the expression of various adipogenic genes, in the latter stage [39]. In contrast, there is a notable increase in the expression levels of these factors in differentiated adipocytes. Recent studies have shown that probiotics can reduce the expression of various adipogenesis-related transcription factors, including CEBPα, in 3T3-L1 cells and animal models [30, 40, 41]. In this study, LRCC5314 significantly reduced CEBPα, PPARγ, adiponectin, and FAS levels in differentiation-induced 3T3-L1 cells and indirectly reduced the TNF-α and IL-6 levels. Since the efficacy of LRCC5314 in regulating adipogenesis-related transcription factors was confirmed along with a reduction in lipid droplet production, additional follow-up studies are needed to identify the efficacy of LRCC5314 in alleviating or preventing obesity.

Cortisol is a hormone that responds to stress such as strenuous exercise by activating fat utilization, facilitated via the decomposition of free fatty acids. Although normal cortisol levels may help the body respond to stress, high cortisol levels in the blood can cause negative nitrogen equilibrium in the body, resulting in a loss of immunity [42]. In this study, LRCC5314 showed an approximate 35% reduction in cortisol levels in H295R cells under conditions of stress. Thus, further studies are needed to identify whether such a reduction of the cortisol levels is also exhibited in vivo in animal models and human trials, and to explore the associated alleviation of stress.

In conclusion, the present study assessed the probiotic properties of LRCC5314 newly isolated from kimchi. LRCC5314 showed high acid tolerance, bile acid tolerance, and adhesion activity, suggesting a high likelihood that numerous viable cells would reach the intestine and adhere to the intestinal surface. LRCC5314 also exhibited other functionalities of a probiotic, including the ability to inhibit the activities of α-amylase and α-glucosidase, which are involved in the elevation of postprandial blood glucose, further suggesting its potential use in the prevention and/or management of diabetes. Moreover, when administered to LPS-stimulated RAW 264.7 cells, LRCC5314 was remarkably effective in reducing the levels of inflammatory cytokines such as IL-1β and NO. When administered to differentiating 3T3-L1 preadipocytes, this new LAB strain reduced the number of lipid droplets and TG levels while also inhibiting the expression of various adipogenesis-related transcription factors. Collectively, these results demonstrate the potential use of LRCC5314 in the alleviation and prevention of obesity. If the efficacy and mechanisms of these probiotic properties, confirmed in vitro, can be validated through further studies in animals and humans, LRCC5314 could be developed as a probiotic with excellent combined efficacy and pharmabiotic potential.

## Figures and Tables

**Table 1:** Acid and bile tolerance, adhesion, and enzyme inhibition activities of LRCC5314 and related representative isolated strains in the genus *L. plantarum*.

Strains	Tolerance (%)		Adhesion (%)	Enzyme-activity inhibition activity (%)	
	
	Acid	Bile		α-amylase	α-glucosidase
*L. plantarum* LRCC5314	110.4 ± 2.3****	101.3 ± 0.9	89.9 ± 2.6	72.9 ± 0.9****	51.2 ± 0.3****
*L. plantarum* KCTC 3108^T^	91.9 ± 0.7	99.7 ± 0.9	87.3 ± 2.1	47.4 ± 1.6	15.8 ± 1.0
*L. plantarum* KCTC 3099	81.9 ± 1.0***	96.7 ± 0.6	86.9 ± 1.0	64.6 ± 1.5****	23.3 ± 1.7**
*L. plantarum* LRCC5193	93.7 ± 0.4	99.8 ± 0.7	92.0 ± 1.1	21.8 ± 0.7****	12.2 ± 1.1
*L. plantarum* LRCC5226	112.4 ± 1.5****	102.3 ± 0.7	85.4 ± 2.5	5.1 ± 1.7****	1.4 ± 0.3****
*L. plantarum* LRCC5230	81.5 ± 1.0***	97.5 ± 0.6	84.8 ± 0.9	32.8 ± 1.8****	8.0 ± 0.3**
*L. plantarum* LRCC5273	110.8 ± 2.1****	104.0 ± 0.1**	82.6 ± 1.1	15.6 ± 2.1****	11.2 ± 1.1
*L. plantarum* LRCC5277	84.1 ± 1.1**	96.6 ± 1.0*	81.6 ± 0.4	10.8 ± 0.3****	3.0 ± 0.9****
*L. plantarum* LRCC5309	103.8 ± 1.7****	101.6 ± 0.6	87.6 ± 1.5	68.9 ± 0.8****	16.0 ± 1.7
*L. plantarum* LRCC5310	106.2 ± 1.4****	101.2 ± 0.9	92.2 ± 0.8	62.2 ± 1.0****	44.7 ± 2.1****

Results are expressed as mean ± SE (N = 3) and *p*-values are shown as * *p* < 0.05 ** *p* < 0.005, *** *p* < 0.0005, **** *p* < 0.0001.

Statistical differences from comparison with *L. plantarum* KCTC 3108^T^, are marked with *.

**Table 2:** Utilization of carbohydrates by LRCC5314.

Carbohydrates	Results	Carbohydrates	Results	Carbohydrates	Results
Control	-	α-Methyl-D-Mannoside	-	Turanose	+
Glycerol	-	α-Methyl-D-Glucoside	-	Lyxose	-
Erythritol	-	N-Acetyl-Glucosamine	+	Tagatose	-
D-Arabinose	-	Amygdalin	+	D-Fucose	-
L-Arabinose	+	Arbutin	+	L-Fucose	-
Ribose	-	Esculin	+	D-Arabitol	-
D-Xylose	-	Salicin	+	L-Arabitol	-
L-Xylose	-	Cellobiose	+	Gluconate	-
Adonitol	-	Maltose	+	2-Ketone-Gluconate	-
β-methyl-D-Xylose	-	Lactose	+	5-Keto-Gluconate	-
Galactose	+	Melibiose	+		
Glucose	+	Sucrose	+		
Fructose	+	Trehalose	+		
Mannose	+	Inulin	-		
Srobose	-	Melezitose	+		
Rhamnose	-	Raffinose	+		
Dulcitol	-	Starch	-		
Inositol	-	Glycogen	-		
Mannitol	+	Gentiobiose	-		
Sorbitol	+	Gentiobiose	-		

**Table 3:** Manufacture and standardization of LRCC5314 on a commercial scale.

Contents	Batch No.			Average

	1	2	3	
Manufactured (kg)	3.6	3.6	3.7	3.6
Viable cells (log CFU/g)	11.5	11.6	11.6	11.6
Moisture (%)	3.1	2.7	3.2	3.0

## References

[1] Singh K, Kallali B, Kumar A, Thaker V (2001). Probiotics: a review. Asian. Pac. J. Trop. Biomed..

[2] Isolauri E, Sütas Y, Kankaanpää P, Arvilommi H, Salminen S (2001). Probiotics: effects on immunity. Am. J. Clin. Nutr..

[3] Leininger DJ, Roberson JR, Elvinger F (2001). Use of eosin methylene blue agar to differentiate *Escherichia coli* from other gramnegative mastitis pathogens. J. Vet. Diagn. Invest..

[4] Silva M, Jacobus NV, Deneke C, Gorbach SL (1987). Antimicrobial substance from a human *Lactobacillus* strain. Antimicrob. Agents Chemother..

[5] Aoun A, Darwish F, Hamod N (2020). The influence of the gut microbiome on obesity in adults and the role of probiotics, prebiotics, and synbiotics for weight loss. Prev. Nutr. Food Sci..

[6] Mohajeri MH, La Fata GL, Steinert RE, Weber P (2018). Relationship between the gut microbiome and brain function. Nutr. Rev..

[7] Valdes AM, Walter J, Segal E, Spector TD (2018). Role of the gut microbiota in nutrition and health. BMJ.

[8] Casey DE, Haupt DW, Newcomer JW, Henderson DC, Sernyak MJ, Davidson MD (2004). Antipsychotic-induced weight gain and metabolic abnormalities: implications for increased mortality in patients with schizophrenia. J. Clin. Psychiatry 65 Suppl..

[9] Yadav R, Shukla P (2017). An overview of advanced technologies for selection of probiotics and their expediency: a review. Crit. Rev. Food Sci. Nutr..

[10] Cammarota M, De Rosa M, Stellavato A, Lamberti M, Marzaioli I, Giuliano M (2009). In vitro evaluation of *Lactobacillus plantarum* DSMZ 12028 as a probiotic: emphasis on innate immunity. Int. J. Food Microbiol..

[11] DeVries MC, Vaughan EE, Kleerebezem M, deVos WM (2006). Lactobacillus plantarum survival, functional and potential probiotic properties in the human intestinal tract. Int. Dairy J..

[12] Scholz E (1984). Karl Fischer. Karl Fischer Titration.

[13] Lim JH, Yoon SM, Tan PL, Yang S, Kim SH, Park HJ (2018). Probiotic properties of *Lactobacillus plantarum* LRCC5193, a plant-origin lactic acid bacterium isolated from Kimchi and its use in chocolates. J. Food Sci..

[14] Sookkhee SM, Chulasiri M, Prachyabrued W (2001). Lactic acid bacteria from healthy oral cavity of Thai volunteers: inhibition of oral pathogens. J. Appl. Microbiol..

[15] Strahinic I, Busarcevic M, Pavlica D, Milasin J, Golic N, Topisirovic L (2007). Molecular and biochemical characterizations of human oral *lactobacilli* as putative probiotic candidates. Oral Microbiol. Immunol..

[16] Bosch M, Rodriguez M, Garcia F, Fernández E, Fuentes MC, Cune J (2012). Probiotic properties of *Lactobacillus plantarum* CECT 7315 and CECT 7316 isolated from faeces of healthy children. Lett. Appl. Microbiol..

[17] Lane D, Stackebrandt E, Goodfellow M (1994). 16S/23S rRNA sequencing. Nucleic Acid Techniques in Bacterial Systematics.

[18] Xiao Z, Storms R, Tsang A (2006). A quantitative starch-iodine method for measuring alpha-amylase and glucoamylase activities. Anal. Biochem..

[19] Shai LJ, Magano SR, Lebelo SL, Mogale AM (2011). Inhibitory effects of five medicinal plants on rat alpha-glucosidase: comparison with their effects on yeast alpha-glucosidase. J. Med. Plants Res..

[20] Mosmann T (1983). Rapid colorimetric assay for cellular growth and survival: application to proliferation and cytotoxicity assays. J. Immunol. Methods.

[21] Robbins KS, Greenspan P, Pegg RB (2016). Effect of pecan phenolics on the release of nitric oxide from murine RAW 264.7 macrophage cells. Food Chem..

[22] Sakuma S, Sumida M, Endoh Y, Kurita A, Yamaguchi A, Watanabe T (2017). Curcumin inhibits adipogenesis induced by benzyl butyl phthalate in 3T3-L1 cells. Toxicol. Appl. Pharmacol..

[23] Dusch H, Altwegg M (1995). Evaluation of five new plating media for isolation of *Salmonella* species. J. Clin. Microb..

[24] Saarela M, Mogensen G, Fondén R, Mättö J, Mattila-Sandholm T (2000). Probiotic bacteria: safety, functional and technological properties. J. Biotechnol..

[25] Mcdonald LC, Fleming HP, Hassan HM (1990). Acid tolerance of *Leuconostoc mesenteroides* and *Lactobacillus casei*. Appl. Environ. Microb..

[26] Gilliland SE, Staley TE, Bush LJ (1984). Importance of bile tolerance of *Lactobacillus acidophilus* used as a dietary adjunct. J. Dairy Sci..

[27] Salminen SE, Isolauri E, Salminen E (1996). Clinical uses of probiotics for stabilizing the gut mucosal barrier: successful strains and future challenges. Antonie Van Leeuwenhoek.

[28] Bernet MF, Brassart D, Neeser JR, Servin A (1994). *Lactobacillus acidophilus* LA 1 binds to cultured human intestinal cell lines and inhibits cell attachment and cell invasion by enterovirulent bacteria. Gut.

[29] Ali H, Houghton PJ, Soumyanath A (2006). Alpha-amylase inhibitory activity of some Malaysian plants used to treat diabetes; with particular reference to *Phyllanthus amarus*. J. Ethnopharmacol..

[30] Kinariwala D, Panchal G, Sakure A, Hati S (2020). Exploring the potentiality of *Lactobacillus* cultures on the production of milkderived bioactive peptides with antidiabetic activity. Int. J. Pept. Res. Ther..

[31] Kimoto H, Mizumachi K, Okamoto T, Kurisaki J (2004). New *Lactococcus* strain with immunomodulatory activity: enhancement of Th1-type immune response. Microbiol. Immunol..

[32] Laskin DL (2009). Macrophages and inflammatory mediators in chemical toxicity: a battle of forces. Chem. Res. Toxicol..

[33] Oh NS, Joung JY, Lee JY, Kim Y (2018). Probiotic and anti-inflammatory potential of *Lactobacillus rhamnosus* 4B15 and *Lactobacillus gasseri* 4M13 isolated from infant feces. PLoS One.

[34] Weninger W, von Andrian UH (2003). Chemokine regulation of naïve T cell traffic in health and disease. Semin. Immunol..

[35] Frayn KN, Karpe F, Fielding BA, Macdonald IA, Coppack SW (2003). Integrative physiology of human adipose tissue. Int. J. Obes. Relat. Metab. Disord..

[36] Mishra AP, Sharifi-Rad M, Shariati MA, Mabkhot YN, Al-Showiman SS, Rauf A (2018). Bioactive compounds and health benefits of edible Rumex species-a review. Cell. Mol. Biol..

[37] Cowherd RM, Lyle RE, McGehee RE (1999). Molecular regulation of adipocyte differentiation. Semin. Cell. Dev. Biol..

[38] Rosen ED, Walkey CJ, Puigserver P, Spiegelman BM (2000). Transcriptional regulation of adipogenesis. Genes Dev..

[39] Cornelius POA, MacDougald OA, Lane MD (1994). Regulation of adipocyte development. Annu. Rev. Nutr..

[40] Kang CH, Jeong Y, Han SH, Kim JS, Kim YG, Park HM (2018). In vitro probiotic evaluation of potential antiobesity lactic acid bacteria isolated from human vagina and shellfish. Kor. Soc. Biotech. Bioeng J..

[41] Sorrenti V, Randazzo CL, Caggia C, Ballistreri G, Romeo FV, Fabroni S (2019). Beneficial effects of pomegranate peel extract and probiotics on pre-adipocyte differentiation. Front. Microbiol..

[42] Hoffman JR, Falk B, Radom-Isaac S, Weinstein Y, Magazanik A, Wang Y (1997). The effect of environmental temperature on testosterone and cortisol responses to high intensity, intermittent exercise in humans. Eur. J. Appl. Physiol. Occup. Physiol..

